# Minimally invasive vs. conventional mitral valve surgery: a meta-analysis of randomised controlled trials

**DOI:** 10.3389/fcvm.2024.1437524

**Published:** 2024-08-12

**Authors:** Aamir Amin, Rajanikant Kumar, Shiva Seyed Mokhtassi, Abdullah K. Alassiri, Agatha Odaman, Muhammad Ahmad Raza Khan, Shashi Lakshmana, Zahir Ud Din, Pawan Acharya, Huzaifa Ahmad Cheema, Abdulqadir J. Nashwan, Arsalan Ali Khan, Awab Hussain, Sunil Bhudia, Royce P. Vincent

**Affiliations:** ^1^ Department of Cardiothoracic Surgery, Harefield Hospital, Guy’s and St Thomas’ NHS Foundation Trust, London, United Kingdom; ^2^ Department of Cardiothoracic and Vascular Surgery, Jay Prabha Medanta Super Specialty Hospital, Patna, India; ^3^ Faculty of Medicine, King Abdulaziz University, Jeddah, Saudi Arabia; ^4^ Department of Cardiothoracic Surgery, Royal Brompton Hospital, Guy’s and St Thomas’ NHS Foundation Trust, London, United Kingdom; ^5^ Department of Radiology, Shaukat Khanum Memorial Cancer Hospital, Lahore, Pakistan; ^6^ Department of Surgery, Khyber Medical College, Peshawar, Pakistan; ^7^ Lister Hospital, Stevenage, United Kingdom; ^8^ Department of Cardiology, King Edward Medical University, Lahore, Pakistan; ^9^ Hamad Medical Corporation, Doha, Qatar; ^10^ Department of Cardiothoracic Surgery, Rush University Medical Center, Chicago, IL, United States; ^11^ Department of Cardiac Surgery, Yale University School of Medicine, New Haven, CT, United States; ^12^ Department of Clinical Biochemistry, King’s College Hospital NHS Foundation Trust, London, United Kingdom; ^13^ Faculty of Life Sciences & Medicine, King’s College London, London, United Kingdom

**Keywords:** minimally invasive, minithoracotomy, sternotomy, mitral valve surgery, MIMVS

## Abstract

**Objective:**

The evidence underlying the efficacy and safety of minimally invasive mitral valve surgery (MIMVS) is inconclusive. We conducted a meta-analysis to evaluate whether MIMVS improves clinical outcomes compared with conventional sternotomy.

**Methods:**

We searched MEDLINE (via PubMed), Embase, the Cochrane Library, and ClinicalTrials.gov from inception to January 2024 for all randomised controlled trials (RCTs), comparing MIMVS with conventional mitral valve surgery. RevMan 5.4 was used to analyse the data with risk ratio (RR) and mean difference (MD) as the effect measures.

**Results:**

Eight studies reporting data on 7 RCTs were included in our review. There was no significant difference in all-cause mortality, the number of patients requiring blood product transfusion, and the change from baseline in the SF-36 physical function scores between the MIMVS and conventional sternotomy groups. MIMVS reduced the length of hospital stay (MD −2.02 days, 95% CI: −3.66, −0.39) but did not affect the length of ICU stay, re-operation for bleeding, and the incidence of renal injury, wound infection, neurological events, and postoperative moderate or severe mitral regurgitation. MIMVS was associated with a trend toward lower postoperative pain scores (MD −1.06; 95% CI: −3.96 to 0.75).

**Conclusions:**

MIMVS reduced the number of days spent in the hospital and showed a trend toward lower postoperative pain scores, but it did not decrease the risk of all-cause mortality or the number of patients needing blood product transfusions. Further large-scale RCTs are required to inform definitive conclusions, particularly with regard to quality-of-life outcomes investigating functional recovery.

**Systematic Review Registration:**

PROSPERO (CRD42023482122).

## Graphical Abstract

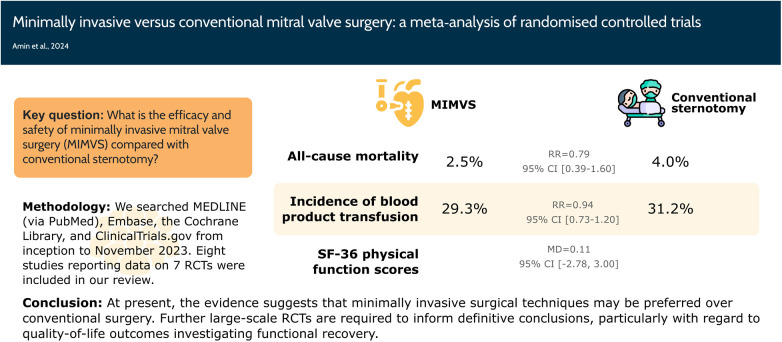

Central Image: Summary of clinical outcomes after minimally invasive mitral valve surgery compared with conventional sternotomy.

## Introduction

First pioneered in the 1990s (1, 2), minimally invasive mitral valve surgery (MIMVS) is a dynamic approach that continues to garner broader acceptance in modern cardiac surgery (3–5). However, it is limited to highly specialised centres across the world that employ a multitude of surgical techniques to minimise operative trauma, including right mini-thoracotomy, partial sternotomy, video-assisted port-access surgery, and even robotic approaches (6–11). Surgeons undergo a variably protracted learning curve to achieve the required competencies despite good mentoring and high-fidelity simulation training (12–14).

Compared with conventional sternotomy, the reported benefits of MIMVS include shorter length of hospital stay, less post-operative pain, less incidence of deep wound infections, use of fewer blood products, and better wound cosmesis (15–18). However, these benefits need to be carefully weighed against a prolonged intraoperative period with increased cross-clamp time and cardiopulmonary bypass time and a reportedly elevated risk of adverse effects such as transient ischaemic attack (TIA), stroke, groin infections, femoral artery injury, and pseudoaneurysm (15, 19, 20).

Despite its ever-growing popularity, the evidence underlying the efficacy and safety of MIMVS is indeterminate as previous systematic reviews on the subject have included non-randomized observational and propensity score matched studies, thus carrying a risk of confounding bias and poor internal validity (18, 21–26). Furthermore, the recent availability of data from a large randomised controlled trial (RCT) has strengthened the evidence base, thus necessitating a systematic re-appraisal of all available data (27).

Therefore, we aimed to conduct a meta-analysis of RCTs to deliberate superior evidence in evaluating whether MIMVS improves clinical outcomes compared with conventional sternotomy in adults requiring surgical intervention for mitral valve disease.

## Methods

This review has been registered in PROSPERO (CRD42023482122). The procedures for conducting this review adhered to the guidelines outlined in the Cochrane Handbook for Systematic Reviews of Interventions (28). Furthermore, the study was reported following the recommendations of the Preferred Reporting Items for Systematic Reviews and Meta-Analysis (PRISMA) statement (29).

### Data sources & search strategy

We comprehensively searched MEDLINE (via PubMed), Embase (via Ovid), Cochrane Central Register of Controlled Trials (CENTRAL, via The Cochrane Library), and ClinicalTrials.gov using a search strategy consisting of relevant keywords and Medical Subject Headings (MeSH) from inception till January 2024. The detailed search strategy is presented in Supplementary Table S1. The search process involved no specific filters or limits. Additionally, we screened the reference lists of the included articles for other relevant studies. A partial search of Google Scholar was also conducted to retrieve any relevant grey literature.

### Eligibility criteria

We included RCTs that compared MIMVS to conventional sternotomy in adults requiring surgery for mitral valve disease. We excluded studies that employed robotic surgery or enhancement, observational studies, quasi-randomized studies, and reviews.

### Study selection

All studies obtained from our online search were imported into Mendeley Desktop 1.19.8, and then duplicates were removed. Two authors independently screened the titles and abstracts, followed by the full texts. A third author was assigned to resolve any conflicts.

### Data extraction

The following data were extracted into a pre-piloted Excel sheet: (1) summary of the included studies (study ID, location, sample size); (2) baseline characteristics of the patients; (3) outcome data of the outcomes. Our primary outcomes were all-cause mortality, number of patients requiring blood product transfusion, and change from baseline in the 36-Item Short Form Health Survey (SF-36) physical functioning percentage scores. Our secondary outcomes were the ICU length of stay, hospital length of stay, post-operative pain scores on the 3rd or 4th day, re-operation for bleeding, renal injury, wound infection, neurological events, and post-operative moderate or severe mitral regurgitation (MR). The extracted data were cross-checked, and any errors were rectified. If two or more reports of the same RCT were found, data from the report with longer follow-up was preferred.

### Risk of bias assessment

Two authors independently assessed the quality of the included studies using the revised Cochrane Risk of Bias tool (RoB 2.0). RoB 2.0 investigates the risk of bias according to five domains: (1) randomisation process; (2) deviations from intended interventions; (3) missing outcome data; (4) outcome measurement; and (5) selection of the reported result.

### Statistical analysis

The meta-analysis was performed using the Review Manager (RevMan) software version 5.4. We used risk ratios (RRs) and mean differences (MDs) along with their corresponding 95% confidence intervals (CIs) to evaluate our outcomes. The random-effects model was employed to pool data using the Mantel-Haenszel approach for dichotomous outcomes and the Inverse Variance approach for continuous outcomes. Heterogeneity was assessed using the *I*^2^ and Chi-square tests; the Chi-square test determined substantial heterogeneity for an alpha level below 0.1, while the *I*^2^ test was interpreted according to the guidance presented in the Cochrane Handbook for Systematic Reviews of Intervention (28). We could not investigate publication bias as the number of included studies was less than 10.

## Results

### Study selection and characteristics

**Figure 1:**
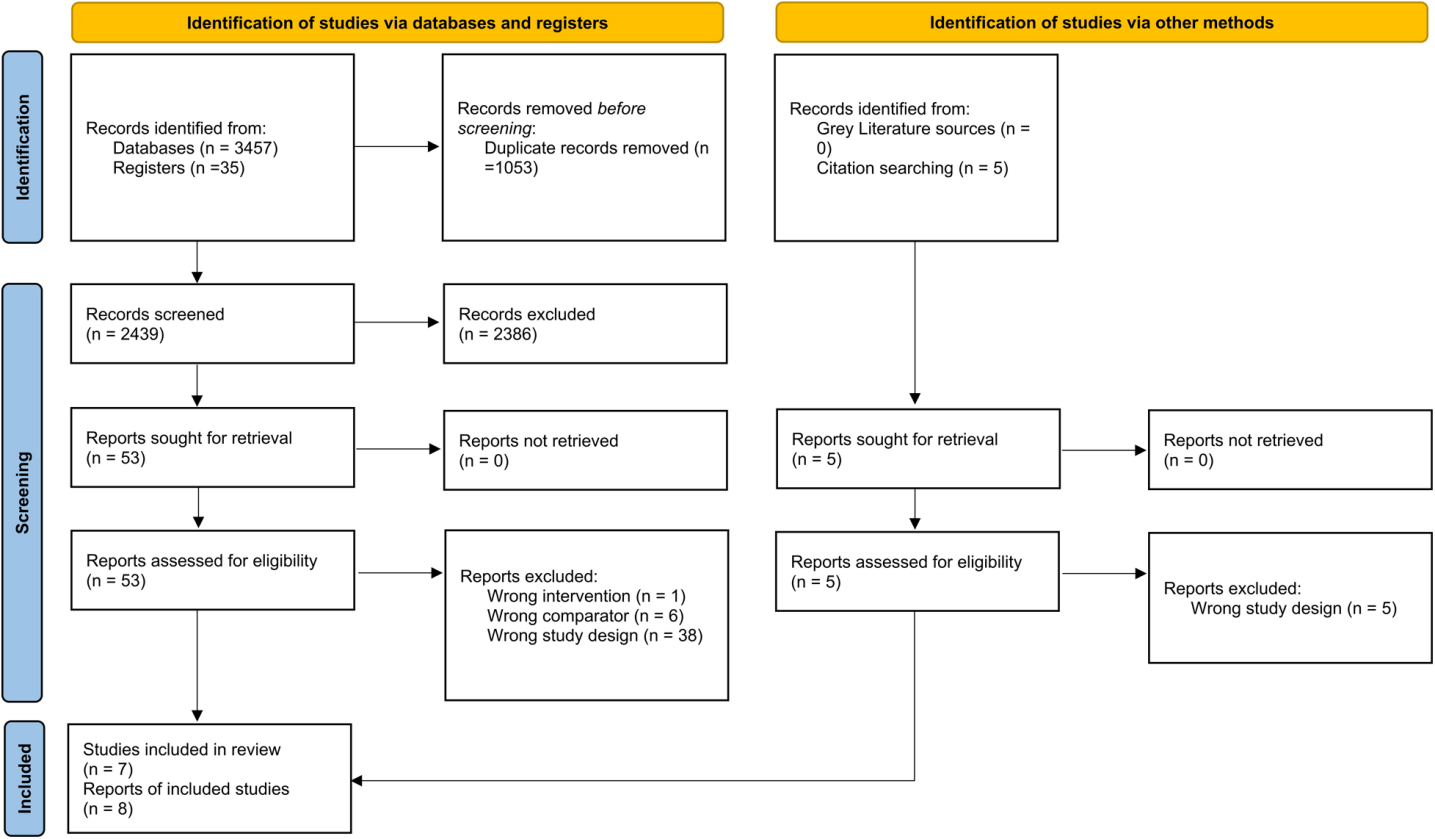
PRISMA 2020 flowchart of the study selection procedure.

Our meta-analysis included eight reports providing data on 7 RCTs (Figure 1) (27, 30–36). Two reports of the same RCT were obtained, one reporting short- and mid-term results (34) and the other reporting long-term results at 3 years of follow-up (36). All were conducted in different countries, with sample sizes ranging from 40 to 330 patients. The duration of operation was higher in the MIMVS group. Table 1 shows the characteristics of the included RCTs in detail, and Table 2 shows the baseline characteristics of patients in these RCTs.

**Table 1:** Characteristics of included studies.

Study ID	Location	No. of patients	Population	Intervention	Operation duration	Follow-up duration
Akowuah et al. (27)	United Kingdom	330 (166 vs. 164)	Adults (≥18 years) with degenerative MR requiring mitral valve repair	Right lateral minithoracotomy	227.7 ± 56.4 vs. 184.3 ± 42.6 min	1 year
Dogan et al. (33)	Germany	40 (20 vs 20)	Forty consecutive adult patients with severe mitral valve disease	Right anterior thoracotomy	253.9 ± 50.3 vs 239.4 ± 55.5 min	NR
El-Fiky et al. (32)	Egypt	100 (50 vs. 50)	Mitral valve patients with MS and/or MR requiring surgery	Right anterolateral minithoracotomy	NR	3 months
Kang et al. (31)	South Korea	100 (50 vs. 50)	Adults >16 years undergoing mitral valve repair.	Right anterolateral thoracotomy	NR	NR
Malik et al. (35)	Pakistan	281 (204 vs. 77)	All patients who were selected for mitral valve replacement in the Department of Cardiovascular Surgery at Lady Reading Hospital	Right anterolateral thoracotomy	92 ± 12 vs 101 ± 14 min	2 years
Nasso et al. (36)	Italy	160 (80 vs 80)	Bi-leaflet prolapse of MV (Barlow disease)	Right minithoracotomy	292 ± 110 vs 249 ± 90 min	3 years
Shah et al. (30)	India	64 (32 vs. 32)	Adult patients with mitral valve disorder requiring mitral valve replacement	Right anterolateral thoracotomy	4.7 ± 0.4 vs. 4.6 ± 0.3 h	NR
Speziale et al. (34)	Italy	140 (70 vs. 70)	Isolated, severe MR due to Barlow's disease with indication to undergo reparative surgery	Right minithoracotomy	296 ± 118 vs 249 ± 91 min	1 year

MR, mitral regurgitation; MS, mitral stenosis.

**Table 2:** Baseline characteristics of patients in included studies.

Study ID	No. of patients	Age (years)	Male (%)	LVEF% - n(%) or EF(%) - mean ± SD	NYHA III/IV – *n* (%) or mean ± SD
Akowuah et al. (27)	330 (166 vs. 164)	67.3 ± 10.1 vs 67.0 ± 11.5	71.1 vs. 68.1	31 to 50 - 40 (24.7) vs. 15 (10.0), 21 to 30 - 1 (0.6) vs. 1 (0.7), > 20 - 1 (0.6) vs 0 (0.0)	III - 53 (31.9) vs 52 (31.9), IV - 11 (6.6) vs 14 (8.6)
Dogan et al. (33)	40 (20 vs 20)	60.1 12.3 vs 63.2 13.6	9.0 vs 10.0	63.4 ± 10.6 vs 65.2 ± 11.6	3 ± 0.3 vs 2.9 ± 0.4
El-Fiky et al. (32)	100 (50 vs. 50)	22 ± 10 vs. 23 ± 9 years	10.0 vs. 14.0	45 ± 8 vs 48 ± 9	2.7 ± 0.6 vs. 2.9 ± 0.8
Kang et al. (31)	100 (50 vs. 50)	50.7 ± 11.8 vs 52.8 ± 16.9	40 vs 48	62.5 ± 7.2 vs 66.5 ± 6.6	NR
Malik et al. (35)	281 (204 vs. 77)	28 ± 11 vs 26 ± 12	26.69 vs 23.37	NR	NR
Nasso et al. (36)	160 (80 vs 80)	53.9 ± 10.6 vs 54.3 ± 10.5	46 vs 45	<50 - 14 (17.5) vs 15 (18.8), <35 - 3 (8.8) vs 2 (2.5)	23 (28.8) vs 20 (25)
Shah et al. (30)	64 (32 vs. 32)	44.41 ± 8.2 vs. 42.56 ± 6.2	40.6 vs. 31.3	<50 - 78.1 vs. 68.7	III - 24 (75) vs 20 (62.5), IV - 8 (25) vs 12 (37.5)
Speziale et al. (34)	140 (70 vs. 70)	53.2 ± 10.4 vs 54 ± 10.1	41 vs 43	<50 - 11 (15.7) vs 12 (17.1)	21 (30) vs.19 (27)

### Risk of bias assessment

**Figure 2:**
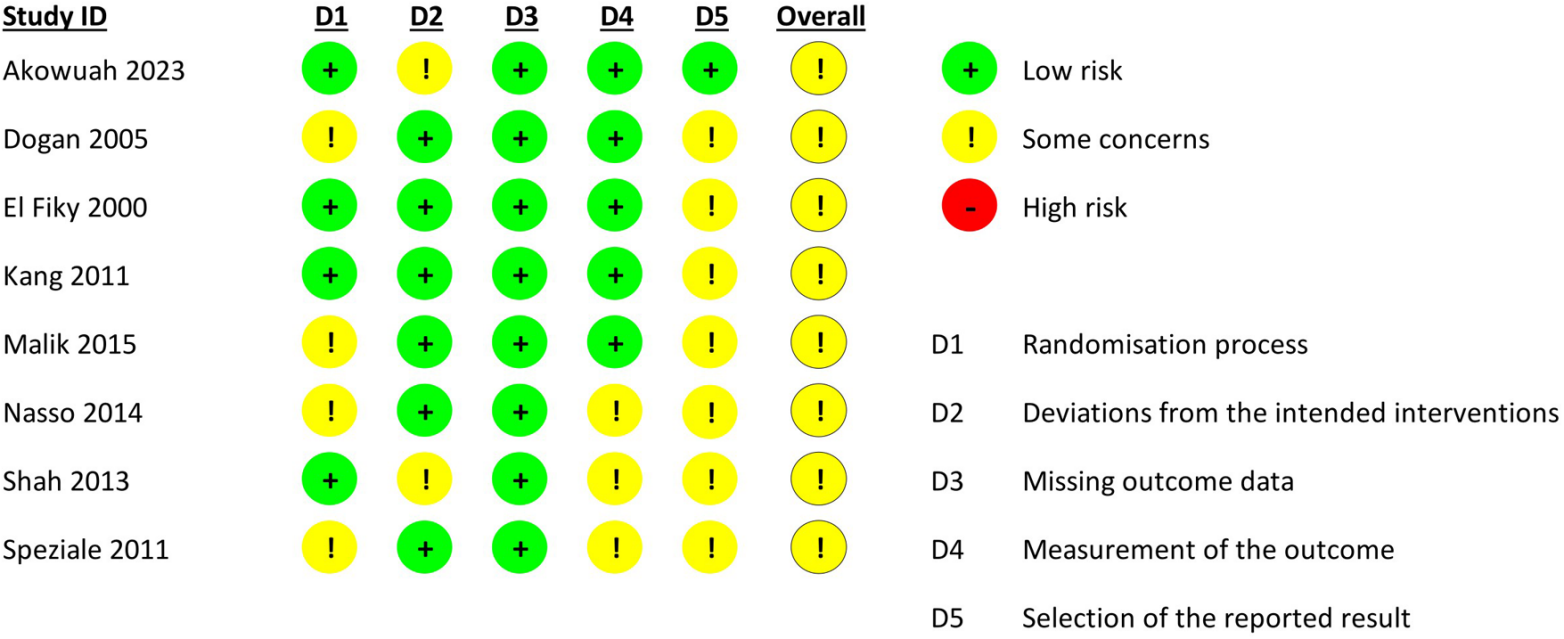
Quality assessment of included trials.

All trials had some concerns of bias, primarily due to the absence of a publicly available protocol and/or issues in the randomisation process or measurement of the outcome domain. Figure 2 illustrates the risk of bias assessment.

## Results of the meta-analysis

### Primary outcomes

**Figure 3:**
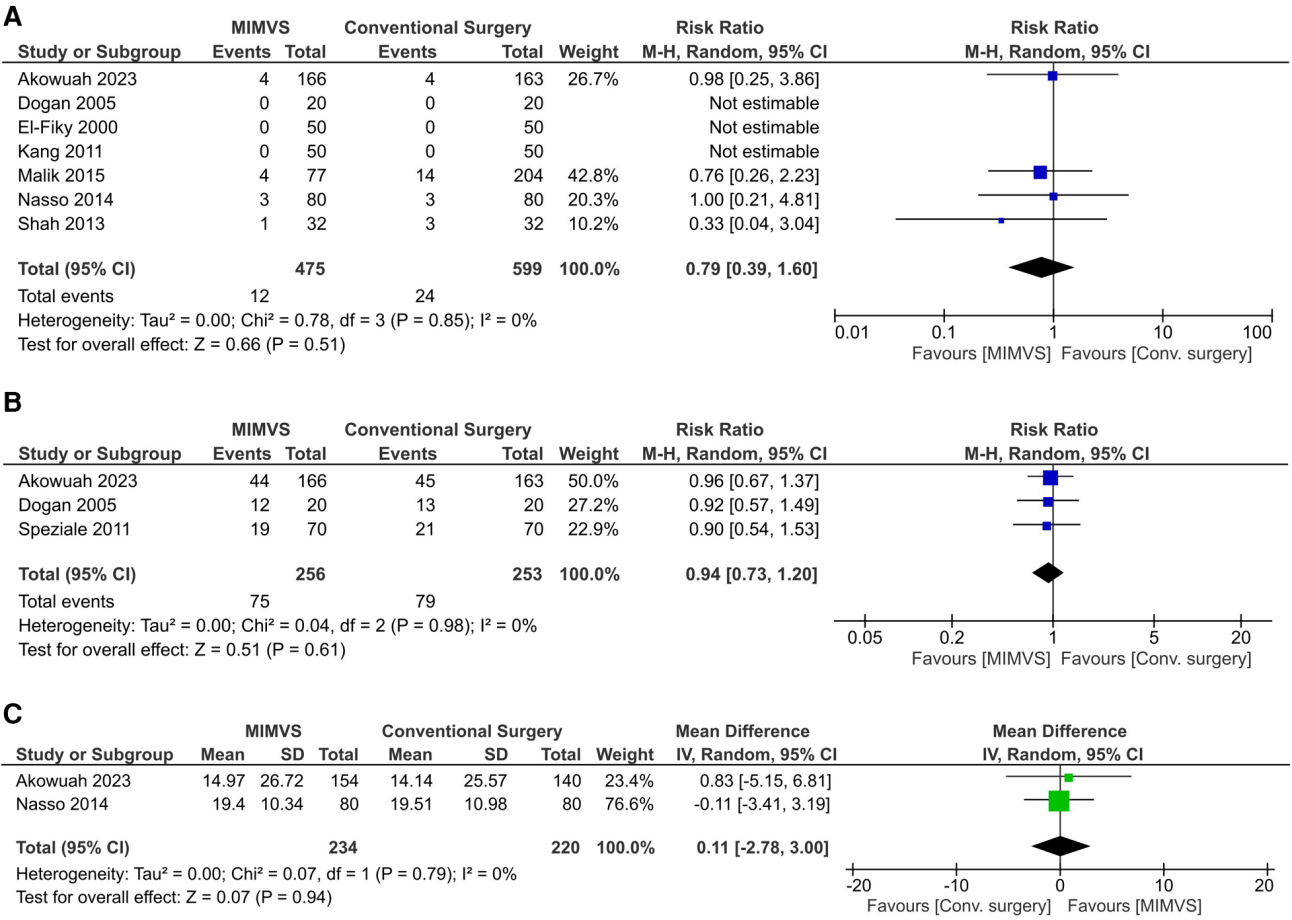
Effect of minimally invasive mitral valve surgery compared to conventional sternotomy on: A) all-cause mortality; B) the number of patients requiring blood product transfusion; and C) the change from baseline in the 36-item short form health survey (SF-36) physical function percentage scores.

There was no significant difference between the MIMVS and the conventional mitral valve surgery groups in the risk of all-cause mortality (RR 0.79; 95% CI: 0.39–1.60, *I*^2 ^= 0%; Figure 3A) and the number of patients needing blood product transfusion (RR 0.94; 95% CI: 0.73–1.20, *I*^2^ = 0%, Figure 3B).

MIMVS was not associated with statistically significant improvement in the SF-36 physical functioning percentage score from baseline (MD 0.11; 95% CI: −2.78 to 3.00, *I*^2^ = 0%; Figure 3C).

### Secondary outcomes

MIMVS had no significant effect on the ICU length of stay (MD −0.87 days; 95% CI: −2.06 to 0.33 days, *I*^2^ = 99%; Supplementary Figure S1) but reduced the hospital length of stay (MD −2.02 days; 95% CI: −2.02 to −0.39 days, *I*^2^ = 95%; Supplementary Figure S2).

There was no significant difference between the MIMVS and conventional surgery groups in the number of patients who needed re-operation for bleeding (RR 0.52; 95% CI: 0.16–1.65, *I*^2^ = 12%; Supplementary Figure S3). There was a trend towards lower post-operative pain scores on the 3rd or 4th day with MIMVS (MD −1.06; 95% CI: −3.96 to 0.75, *I*^2^ = 98%; Supplementary Figure S4).

There were no significant differences between the two groups in the incidence of renal injury (RR 1.17; 95% CI: 0.44–3.11, *I*^2^ = 0%; Supplementary Figure S5), wound infection (RR 1.21; 95% CI: 0.63- 2.31, *I*^2^ = 0%; Supplementary Figure S6), neurological events (RR 0.99; 95% CI: 0.44–2.22, *I*^2^ = 0%; Supplementary Figure S7), and postoperative moderate or severe MR (RR 0.95; 95% CI: 0.46–1.97, *I*^2^ = 0%; Supplementary Figure S8).

## Discussion

To the best of our knowledge, this is the most comprehensive systematic review and meta-analysis to date that evaluates minimally invasive techniques compared with conventional sternotomy for mitral valve surgery using data from RCTs only. Our analysis showed that the two procedures had similar all-cause mortality and blood product transfusion rates. Compared to conventional sternotomy, MIMVS did not improve physical functioning as assessed by SF-36. MIMVS reduced the length of hospital stay but did not affect the ICU length of stay, re-operation for bleeding, and the incidence of renal injury, wound infection, neurological events, and postoperative moderate or severe MR. There was a trend towards lower postoperative pain scores with MIMVS.

Our findings contrast with several prior reviews, which have demonstrated that MIMVS improves postoperative clinical outcomes, including mortality, need for blood transfusions, and/or renal failure (22, 25). Conversely, our meta-analysis reaffirms the findings of other recent systematic appraisals, which have shown similar outcomes with the two surgical techniques apart from a shorter hospital stay in the MIMVS group (23, 26). However, the past reviews suffered from many shortcomings, with the primary one being the reliance on data from primarily observational and single-armed studies, thus incurring a high risk of confounding bias. One previous meta-analysis focused on RCTs but provided low-quality evidence due to the availability of only a few small trials (37). There is a paucity of randomised controlled data, but the recently published UK Mini Mitral Trial, the largest trial on this topic to date (27), has made significant strides toward addressing this knowledge gap. A recent large meta-analysis included 8 RCTs in its analysis, but out of those, one was an observational study mistakenly classified as an RCT (38), and two were different reports of the same RCT (34, 36); the inclusion of multiple reports of a single RCT in a single analysis duplicates patient data and may lead to erroneous and skewed results (28). Our systematic review addresses many of the limitations of previous analyses by including only RCTs, data from one report of an RCT out of multiple ones in each analysis as recommended by the Cochrane guidelines, and data from the UK Mini Mitral Trial (27), which has not been collated in a systematic appraisal thus far. Consequently, our meta-analysis provides more reliable results and a clearer and more robust understanding of this topic.

One of the concerns about MIMVS has been a greater postoperative risk of stroke based on data from observational studies (18, 39, 40); however, we did not find an elevated rate of neurological events in our analysis, thus corroborating the results of the UK Mini Mitral Trial (27). Additionally, there was no increase in any of the other evaluated adverse events, including wound infection, renal injury, and postoperative MR, further allaying any safety and quality of repair concerns. MIMVS has reportedly been associated with aortic dissection; however, we could not assess this postoperative complication due to a scarcity of data. Future RCTs should also evaluate the occurrence of aortic dissection to provide conclusive proof.

The primary benefits of minimally invasive surgery are usually seen with faster functional recovery and lesser postoperative pain. However, surprisingly, very few studies and, subsequently, systematic reviews based on these studies have attempted to assess the quality-of-life outcome measures. In our review, we sought to address this issue and pool relevant data from RCTs. We found no improvement in physical functioning according to SF-36, but we did observe a trend toward lower pain scores in the MIMVS group. Given that data from only two trials was available for these outcomes, and the SF-36 is a generic health survey questionnaire not specific for any surgical technique, new large-scale RCTs focusing on quality of life and patient-reported outcomes assessed through specific tools might reveal the expected benefits of MIMVS.

The evidence from our meta-analysis, taken as a whole, suggests that MIMVS might be preferred over conventional sternotomy due to shorter hospital stays and a possible benefit in functional recovery. Nevertheless, the similar rates of blood transfusion and postoperative complications, such as wound infection, between the two approaches should be highlighted because these have historically been associated with sternotomy access. Therefore, our findings should be interpreted cautiously, and the need for further large-scale RCTs comparing these two techniques should be emphasized before drawing any definitive conclusions.

Some limitations of our meta-analysis need to be stated. Despite the robust analysis, inherent biases were observed across studies, particularly regarding the lack of a registered protocol. Most trials had small sample sizes; therefore, our analysis is likely underpowered for some outcomes, hindering the ability to draw definitive conclusions, especially for the important quality-of-life measures such as the SF-36 physical functioning and postoperative pain scores. Moreover, differences between the trials regarding surgical procedures and postoperative care protocols contribute to heterogeneity. This is an aggregate-level meta-analysis, and we did not have access to individual patient data, limiting our ability to explore any potential effect modifiers. Finally, the short follow-up times of the RCTs preclude evaluation of the longer-term surgical success of the two approaches.

## Conclusions

In our meta-analysis, MIMVS and conventional sternotomy had similar mortality and blood product transfusion rates. MIMVS was associated with a shorter hospital stay and did not increase the risk of any postoperative complications, including re-operation for bleeding and the incidence of renal injury, wound infection, neurological events, and postoperative moderate or severe MR. MIMVS did not improve SF-36 physical functioning scores but was associated with a trend toward lower postoperative pain scores. At present, the evidence suggests that minimally invasive surgical techniques may be preferred over conventional surgery due to their potential short-term benefits for patients and lack of significant drawbacks from a clinical perspective; however, further large-scale RCTs, particularly investigating meaningful patient-reported and quality-of-life outcomes are needed to consolidate the evidence base and provide definitive conclusions.

## Data Availability

The raw data supporting the conclusions of this article will be made available by the authors, without undue reservation.
