# Supplementary material for: Minimally invasive vs. conventional mitral valve surgery: a meta-analysis of randomised controlled trials
Source: Front Cardiovasc Med. 2024 Aug 12;11:1437524. doi: 10.3389/fcvm.2024.1437524 (PMC11345173; doi:10.3389/fcvm.2024.1437524)
Supplement: Supplementary file 1 [file Datasheet1.docx]

**Supplementary Table 1. Search strategy for Embase (Ovid), which was adapted for other databases.**

1. mitral valve/
2. mitral valv*.ti,ab,kw.
3. (bicuspid adj2 valv*).ti,ab,kw.
4. (left atrioventricular adj2 valv*).ti,ab,kw.
5. mitral valve regurgitation/ or ischemic mitral valve regurgitation/
6. (mitral incompetence or mitral insufficiency or mitral regurgitation or ischemic mitral valve regurgitation).ti,ab,kw. 7. mitral valve stenosis/
8. mitral stenos?s.ti,ab,kw.
9. or/1-8
10. thoracotomy/
11. (thoracotom* or mini-thoracotom*).ti,ab,kw.
12. mini-sternotom*.ti,ab,kw.
13. minimally invasive surgery/
14. thoracoscopy/
15. video assisted thoracoscopic surgery/
16. video assisted thoracoscopic surger*.ti,ab,kw.
17. (minimally-invasive or minimal access or thoracoscop*).ti,ab,kw.
18. robotics/
19. Robotic Surgical Procedures/
20. robotic surg*.ti,ab,kw.
21. or/10-20
22. 9 and 21

**Supplementary Figure 1.** Effect of minimally invasive mitral valve surgery compared to conventional sternotomy on ICU length of stay.

**
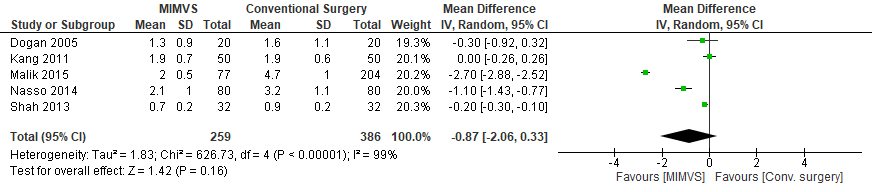
**

**Supplementary Figure 2.** Effect of minimally invasive mitral valve surgery compared to conventional sternotomy on hospital length of stay.

**
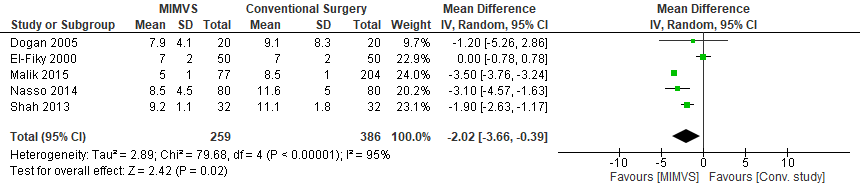
**

**Supplementary Figure 3.** Effect of minimally invasive mitral valve surgery compared to conventional sternotomy on the risk of re-operation for bleeding.

**
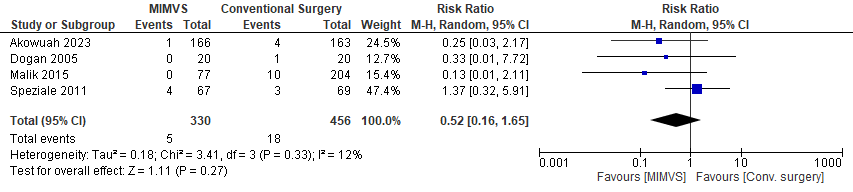
**

**Supplementary Figure 4.** Effect of minimally invasive mitral valve surgery compared to conventional sternotomy on postoperative pain scores on 4^th^ or 5^th^ day.

**
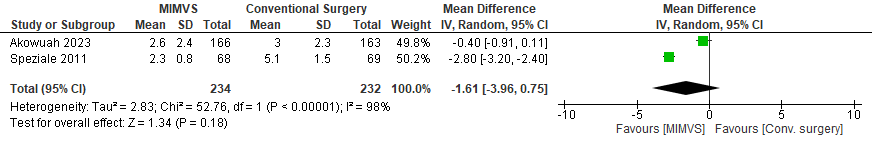
**

**Supplementary Figure 5.** Effect of minimally invasive mitral valve surgery compared to conventional sternotomy on the incidence of renal injury.

**
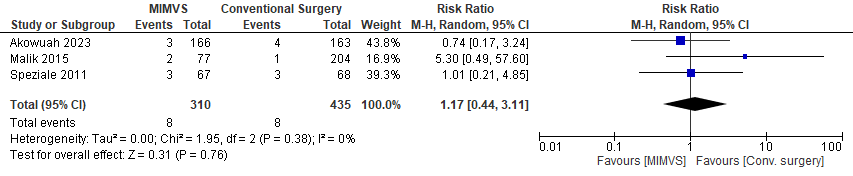
**

**Supplementary Figure 6.** Effect of minimally invasive mitral valve surgery compared to conventional sternotomy on the incidence of wound infection.

**
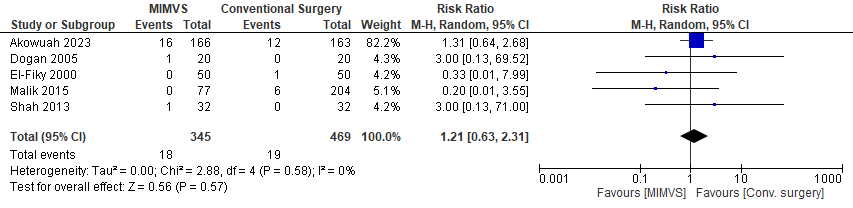
**

**Supplementary Figure 7.** Effect of minimally invasive mitral valve surgery compared to conventional sternotomy on the incidence of neurological events.

**
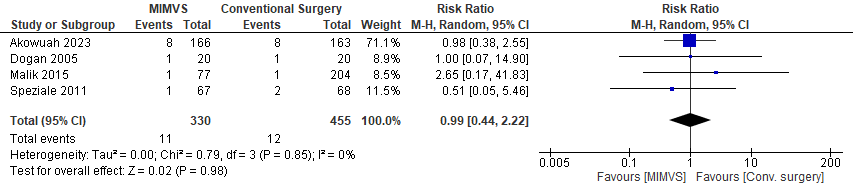
**

**Supplementary Figure 8.** Effect of minimally invasive mitral valve surgery compared to conventional sternotomy on the incidence of postoperative moderate or severe mitral regurgitation.

**
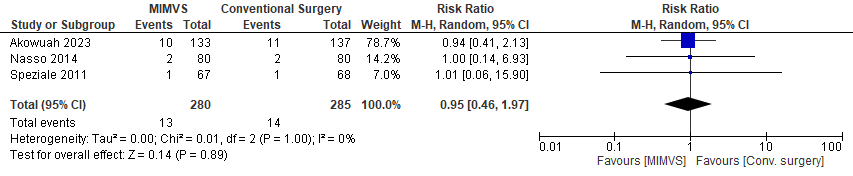
**
